# P-1583. Epidemiological Trends and Risk Factors of STIs Including Syphilis Among Males, LGBT Populations, and Pregnant Women : The Role of Free Health Insurance in resource limit setting

**DOI:** 10.1093/ofid/ofaf695.1762

**Published:** 2026-01-11

**Authors:** shambhu joshi, Dinesh Joshi

**Affiliations:** Mahabaudha Medical Center, Dhangadhi, Seti, Nepal; Mahabaudha Medical Center, Dhangadhi, Seti, Nepal

## Abstract

**Background:**

STIs, including syphilis, are a major public health concern in Nepal, and high prevalence is shown among young males, LGBT populations, and pregnant women. The prevalence of syphilis among attendees of antenatal care is 1.3%, though it was higher among the high-risk groups

**Figure:**
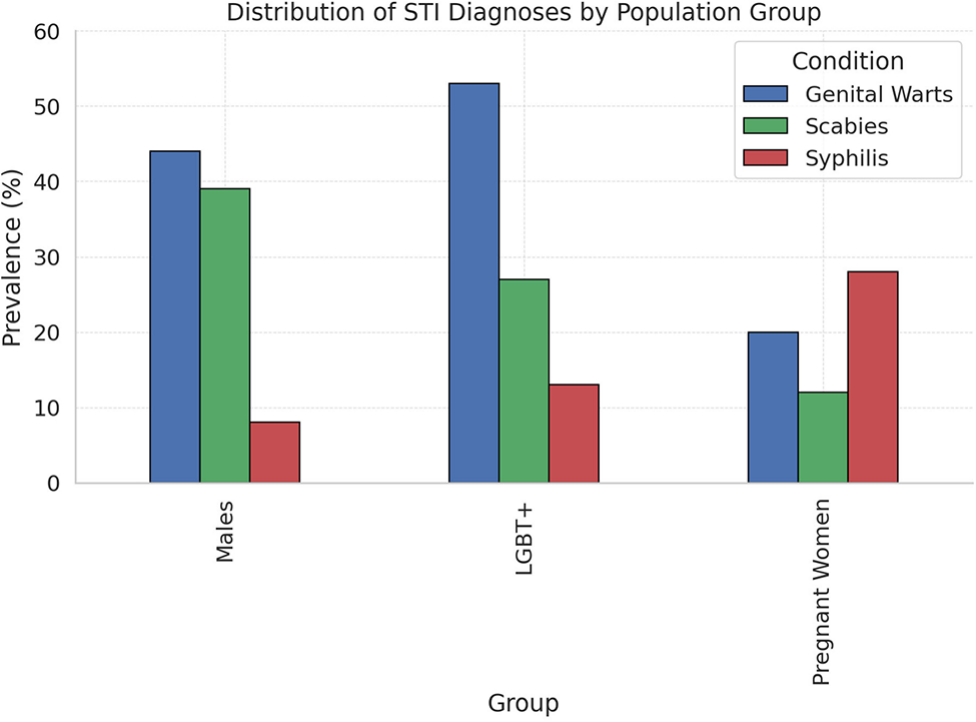
Distribution of STI

**Figure:**
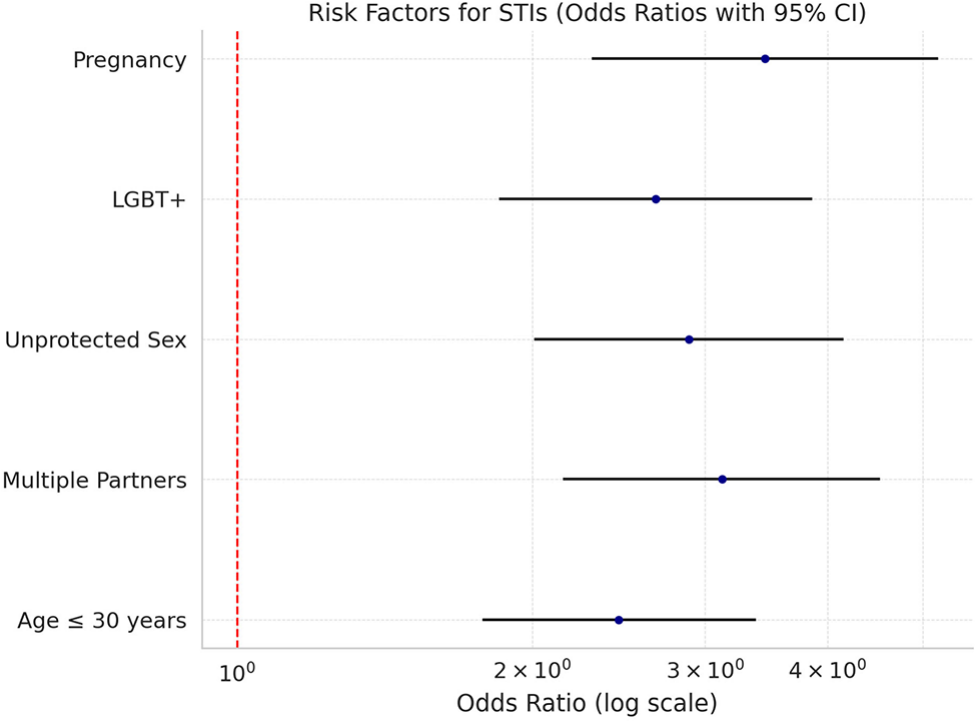
Risk factors in groups

**Methods:**

A cross-sectional study of one year attended were screened for 200 subjects of genital skin lesions and syphilis. Data were analysed with SPSS.

**Results:**

Out of the total number of screened individuals, 156 (3.3%) with genital lesions or serologically positive for syphilis were found. Of these, 116(2.9%) were males, 15(7.5%) were LGBT, and 25 (5%) were pregnant women. Among these, 54% of males (n=63), 73% of LGBT(n=11), and 60% of pregnant women (n=15) were diagnosed to have STIs. Specific conditions included:Genital warts:44% males(n=51), 53% LGBT(n=8), and 20% pregnant women(n=5). Scabies: 39% males(n=45), 27% LGBT(n=4), and 12% pregnant women (n=3). Syphilis: 8% males(n=9), 13% LGBT(n=2), and 28% pregnant women(n=7)

Statistical analysis showed the following significant risk factors that contribute to STIs: Age ≤ 30 years :OR = 2.45, 95% CI: 1.78–3.38, p < 0.001, Having multiple sex partners: OR = 3.12, 95% CI: 2.15–4.52, p < 0.001, History of unprotected sexual exposure: OR = 2.89, 95% CI: 2.01–4.15, p < 0.001, Being LGBT+:OR = 2.67, 95% CI: 1.85–3.86,p < 0.001, Pregnancy: OR = 3.45, 95% CI: 2.30–5.18,p < 0.001.

**Conclusion:**

The study showed the high burden of sexually transmitted infections, especially syphilis, among men, LGBT populations, and pregnant-women. Free laboratory, culture diagnostics, treatment through the Nepal health insurance program mark a significant stride forward in STI prevention and treatment. Acquisition efforts should focus on the reduction of stigma, increase awareness,and expansion via telemedicine. Reach and inclusion into the insurance-program remain critical social determinants to the realization of equity in access to health-care among marginalized populations

**Disclosures:**

All Authors: No reported disclosures

